# Advances in the Microbial Synthesis of 5-Hydroxytryptophan

**DOI:** 10.3389/fbioe.2021.624503

**Published:** 2021-02-03

**Authors:** Xin-Xin Liu, Bin Zhang, Lian-Zhong Ai

**Affiliations:** ^1^Shanghai Engineering Research Center of Food Microbiology, School of Medical Instrument and Food Engineering, University of Shanghai for Science and Technology, Shanghai, China; ^2^College of Bioscience and Bioengineering, Jiangxi Agricultural University, Nanchang, China

**Keywords:** 5-hydroxytryptophan, tryptophan hydroxylase, L-trp, biosynthesis, tetrahydrobiopterin

## Abstract

5-Hydroxytryptophan (5-HTP) plays an important role in the regulation of emotion, behavior, sleep, pain, body temperature, and other physiological functions. It is used in the treatment of depression, insomnia, migraine, and other diseases. Due to a lack of effective biosynthesis methods, 5-HTP is mainly obtained by natural extraction, which has been unable to meet the needs of the market. Through the directed evolution of enzymes and the introduction of substrate supply pathways, 5-HTP biosynthesis and yield increase have been realized. This review provides examples that illustrate the production mode of 5-HTP and the latest progress in microbial synthesis.

## Introduction

**Figure 1:**
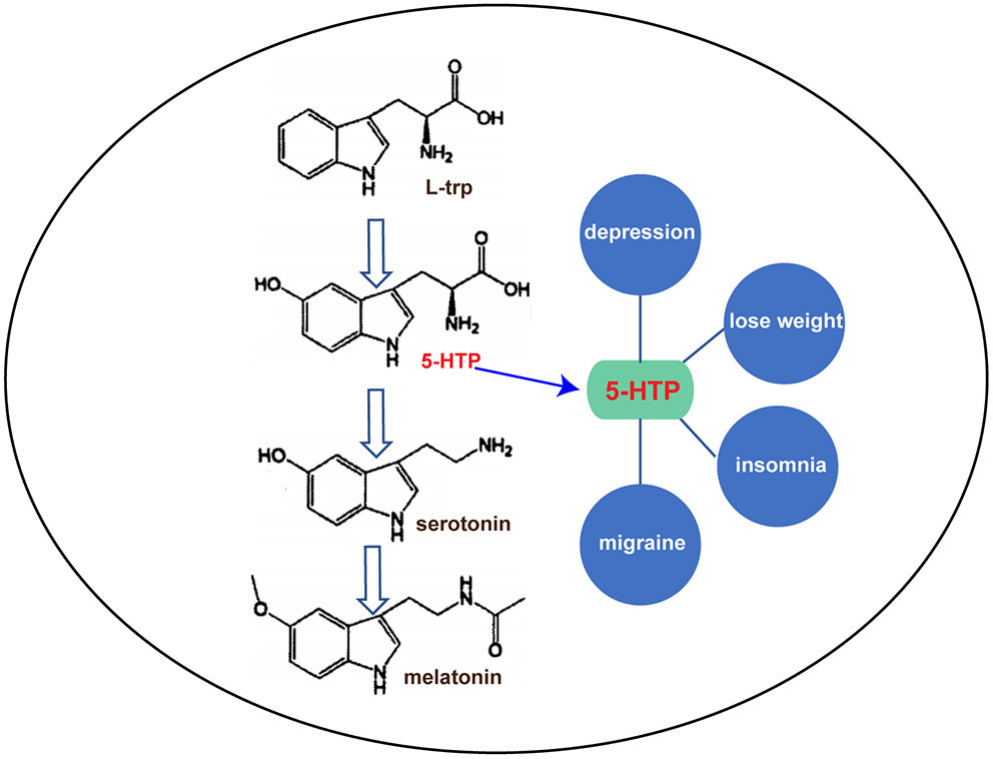
The 5-HTP metabolic pathway and its function.

5-Hydroxytryptophan (5-HTP) is a natural amino acid (AA) that does not participate in protein synthesis. It is derived from tryptophan (trp), and the hydrogen atoms at the 5′-position on the benzene ring of trp are replaced by hydroxyl groups. 5-HTP appears as a fine white powder that is insoluble in water, but is soluble in alcohol. In mammals, 5-HTP is the precursor of the neurotransmitter serotonin and the amine hormone melatonin. It has been successfully used in the treatment of depression, insomnia, migraines, and other diseases due to its regulatory effects on sleep, pain, appetite, and other physiological functions (Figure 1) (Birdsall, 1998). 5-HTP is widely used for psychotropic drugs and weight loss in developed countries. Health-care drugs with 5-HTP as the main ingredient are used in 20 countries. To date, 44 preparation types have been developed worldwide mainly in the form of capsules with 100–150 mg content, as well as in the form of tablets, powders, and sustained-release agents (JunDe et al., 2014). According to the 2014 Thomson Reuters market research report, the annual global sales of 5-HTP were $7.5 million, an increase of 50% over the same period the previous year, with an annual consumption of 1.6 tons.

At present, 5-HTP is mainly extracted from natural products, specifically from the seeds of the African plant *Griffonia Simplicifolia*, but this method of extraction is unable to meet the market demand due to high cost and a lack of raw materials. Chemical synthesis does not depend on natural products. However, it is not currently possible to synthesize 5-HTP economically and effectively due to the tedious steps involved and harsh conditions required. With the development of biotechnology in bioinformatics, genetics, metabolic engineering, biochemistry, protein engineering, and so on, new strategies are available for the use of microorganisms to synthesize 5-HTP. The production of 5-HTP by the microbial method has the advantages of short production cycle, continuous production and mild reaction conditions. Among the organisms used for this method, *Escherichia coli* is a model strain of prokaryotes with clear genetic information and well-established fermentation conditions, making it particularly well-suited for use as a host cell for the study of 5-HTP biosynthesis.

## Physiological Function of 5-HTP

As shown in Figure 1, 5-HTP has been widely studied for its important role in the treatment of depression and for weight loss. Depression is a common mental disorder with high levels of disability and stress. More than 350 million people worldwide suffer from depression, and ~1 million people commit suicide due to depression every year (World Health Organization, 2017). It has become the second most important disease worldwide, posing a serious burden on human beings. Dysfunction of serotonin in the brain is thought to be a major cause of depression. 5-HTP is a natural and safe antidepressant because it can increase serotonin levels in the brain. In a clinical trial of 107 patients with depression, 69% of symptoms improved through the daily intake of 50–300 mg of 5-HTP. The response rate to this drug was significantly faster than that of ordinary drugs (Sano, 1972). In addition, the content of 5-hydroxyindoleacetic acid (a serotonin-decomposition product) in the cerebrospinal fluid of the patients significantly increased after 5-HTP intake, indicating that 5-HTP was successfully converted to serotonin after entering the central nervous system (Takahashi et al., 1975).

Dieting leads to a sharp decrease in serotonin levels in the serum and brain, and a decrease in serotonin leads to gluttonous gluttony. 5-HTP can prevent dieting induced decrease in serotonin in patients with obesity, thus reducing appetite and assisting with weight loss (Ceci et al., 1989; Cangiano et al., 1991, 1992). In addition, 5-HTP can also improve the symptoms of fibromyalgia, including pain, morning stiffness, anxiety, and fatigue (Caruso et al., 1990; Sarzi Puttini and Caruso, 1992; Nicolodi and Sicuteri, 1996). Chronic headaches are caused by reduced serotonin levels in the body. 5-HTP has successfully been used to prevent various types of chronic headaches, including migraines, tension headaches, and adolescent headaches (Bono et al., 1982; Longo et al., 1984; Benedittis and Massei, 1985; Titus et al., 1986; De Giorgis et al., 1987; Maissen and Ludin, 1991; Nicolodi and Sicuteri, 1996). In addition, 5-HTP can increase the rapid eye movement sleep period to improve sleep quality for the treatment of insomnia.

## Production of 5-HTP

The anabolic-metabolic engineering design for 5-HTP has gained considerable attention and has been widely studied because of its important physiological functions and huge market demand. At present, the main methods for producing 5-HTP are natural product extraction, chemical synthesis, and microbial fermentation. Among these methods, natural product extraction remains the primary method for the commercial production of 5-HTP. While microbial synthesis and catalysis provide a fast and environmentally friendly alternative to produce natural compounds of medical value.

### Natural Extraction of 5-HTP

5-HTP is widely present in the seeds of legumes, with the seeds of African plant *Griffonia Simplicifolia* haing the highest content. The leaves and seeds of the Ghanaian tree have been used as medicine in Africa since ancient times to treat wounds, kidney disease, and for enemas. The paste made from its bark is also used to treat skin diseases, in which the main active ingredient is 5-HTP. Lemaire and Adosraku extracted 5-HTP using the alcohol method, and found that it constituted 20.83% of the fresh weight of the seeds (Lemaire and Adosraku, 2010). After optimization of the extraction temperature, the content of 5-HTP in the extract increased from 6.37 to 8.98% and the purity reached 92% (Addotey, 2009). Using ultrafiltration membrane separation technology, the 5-HTP transfer rate was found to be 83.5%, and purity reached 90.5% (Qin et al., 2014). In addition, high-purity 5-HTP was also obtained from flower beans by ultrasonic method (Duan et al., 2017).

However, the natural source of 5-HTP is relatively singular. There is a shortage of raw materials and costs are continually increasing with the increase of exploitation. The increasing market demand is impossible to meet through natural extraction alone. Therefore, there has recently been an increasing number of studies regarding the production of 5-HTP via chemical synthesis and biological fermentation.

### Chemical Synthesis of 5-HTP

5-HTP is synthesized by Michael addition reaction using 5-bromoindole 3-bromo-2-hydroxyimino-propionate as the substrate. The addition reaction connects the side chain of 2-hydroxyimino-propionate to the 3-position of the indole. Subsequently, the reduction reaction, hydrolysis reaction, and resolution steps are introduced to obtain 5-HTP (Fuchun et al., 2013). In an invention patent, Raney-Ni and ZnO were added to an autoclave as catalysts. Trp, glycolic acid, and hydrochloric acid were reacted in the autoclave for 0.25–6 h, then filtered and dried to obtain poly-hydroxy-trp drying substance. The dried substance was then reacted with trp, sodium hydroxide, and deionized water. The 5-HTP crystals were finally obtained after filtration, chromatography and cooling crystallization (Rihe, 2010). Wenhui and colleagues first demonstrated the methyl (ethyl) esterification of L-trp to form tryptophan methyl (ethyl) ester hydrochloride. 5-HTP was obtained after desalting, acetylation, redox, deacetylation, and other reactions. After cooling and crystallization, 5-HTP crystals were obtained. The purity of the product was 99.2% and the overall yield was 45% (Wenhui et al., 2013).

The chemical method for the synthesis of 5-HTP is tedious, harsh, and costly. It uses a variety of organic reagents, resulting in serious environmental pollution. Therefore, this method is not suitable for the large-scale production of 5-HTP.

### Biosynthesis of 5-HTP

**Figure 2:**
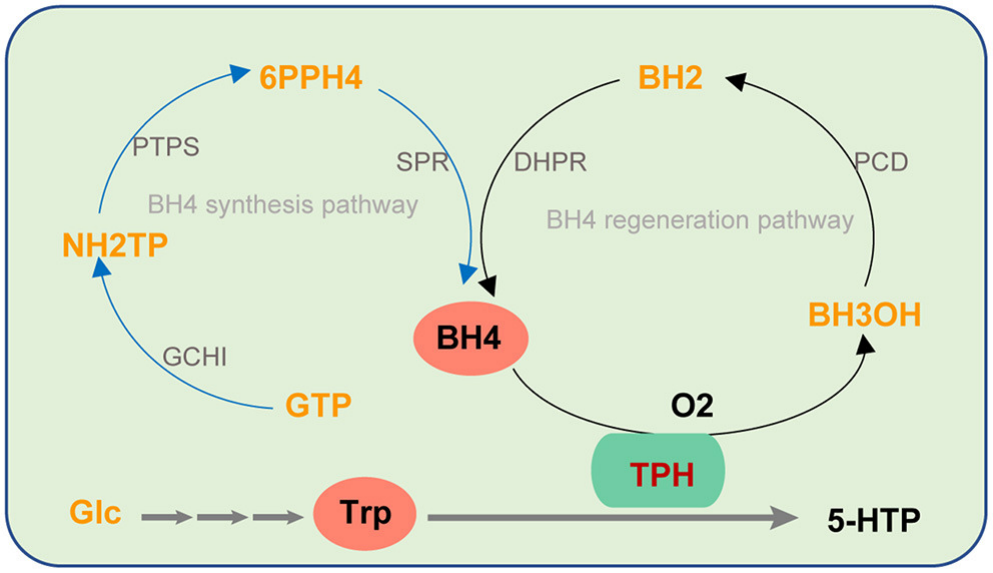
The biosynthesis pathway of 5-HTP catalyzed by TPH using BH_4_ as a cofactor. The blue line represents the BH_4_ synthesis pathway. The black line represents BH_4_ regeneration pathway.

5-HTP production by biological methods is favored due to its advantages, such as a short production cycle, continuous production, and mild reaction conditions. *In vivo*, 5-HTP is obtained by L-trp hydroxylation catalyzed by trp hydroxylase (TPH) using L-trp as a substrate (Figure 2). TPH is a monooxygenase that uses trp and oxygen as substrates, and tetrahydrobiopterin (BH_4_) and Fe^2+^ are required as cofactors in its catalytic process (Kappock and Caradonna, 1996; Fitzpatrick, 1999; Olsson et al., 2010; Roberts and Fitzpatrick, 2013).

## Metabolic Engineering Strategy for 5-HTP Synthesis

*In vivo*, 5-HTP is produced from L-trp and the reaction is catalyzed by TPH, which uses L-trp and O_2_ as substrates and requires BH_4_ and Fe^2+^ as cofactors. The activity of TPH, the supply of L-trp, and the synthesis and regeneration of BH_4_ are three key factors that restrict restricting 5-HTP synthesis.

### L-Tryptophan Hydroxylase (TPH)

TPH, phenylalanine hydroxylase (PAH), and tyrosine Hydroxylase (TH) are pterin-dependent aromatic AA hydroxylases (AAAHs) (Windahl et al., 2008; Olsson et al., 2010). Moreover, these three hydroxylases have substrate interconnectedness, and each hydroxylase can catalyze the hydroxylation of these three aromatic AAs (Olsson et al., 2010; Roberts and Fitzpatrick, 2013). There are two subtypes of TPH in mammals: TPHI and TPH2 (Walther et al., 2003). TPH1 was discovered first and studied in depth. In adults, TPH1 is mainly expressed in non-nerve cells (Murphy et al., 2008). Walther et al. found that the brains of mice could still produce serotonin normally after knocking out the TPH1 gene. Further studies found another TPH, TPH2, in the brains of mice (Walther et al., 2003). TPH1 and TPH2 have 71% AA sequence homology. TPH2 is mainly responsible for the synthesis of serotonin in the central nervous system, including the frontal area, thalamus, hippocampus, amygdala, and hypothalamus, whereas TPH1 is mainly expressed in the pineal gland and the gut and is responsible for the synthesis of serotonin in other parts of the body, such as the heart, lungs, and kidneys (Walther and Bader, 2003; Walther et al., 2003; Patel et al., 2004; Zhang et al., 2004; Sakowski et al., 2006).

First, TPH was heterologous expressed in *E. coli*, and its structure, enzymatic properties, and catalytic mechanism were studied. Windahl et al. (2008) studied the active center structure of chicken TPH1. The Fe^2+^ coordination structure was found to belong to the heme-independent general Fe^2+^ coordination structure. The Fe^2+^ coordination is a distorted trigonal bipyramidal coordination with His273, His278, Glu318, and an imidazole ligand. The substrate trp binds to the hydrophobic pocket of the active center. This hydrophobic pocket is composed of Tyr236, Thr266, Pro267, Glu268, Pro269, His273, Phe314, Phe319, and Lie367 (Windahl et al., 2008). McKinney et al. (2004) expressed human TPH in *E. coli* and *yeast* expression system. They found that the soluble expression of TPH could be enhanced by fusion expression with maltose-binding protein. The fusion expressed TPH's enzyme activity and affinity with L-trp was significantly improved. Moran et al. (1998) expressed rabbit-derived TPH in *E. coli*. It was found that the soluble expression was significantly increased after removing 101 AAs at the N-terminal and 28 AAs at the C-terminal. Interestingly, this mutated TPH exists in the form of a monomer rather than a tetramer. Kino et al. (2009) found that Leu101 and Trp180 from the active center of *Pseudomonas aeruginosa* PAH exerted effects on substrate specificity and hydroxylase activity. The enzyme catalytic rate constant, K_cat_, increased 5.2 times after mutation at these two sites (Kino et al., 2009).

### Synthesis and Regeneration of BH_4_

One difficulty in using *E. coli* to produce 5-HTP is that it does not synthesize the coenzyme BH_4_, which is essential for TPH. *E. coli* can synthesize analogs of BH_4_, tetrahydromonapterin (MH_4_) (Ikemoto et al., 2002). PAH of *Pseudomonas aeruginosa* can use MH_4_ as a coenzyme to hydroxylate L-phenylalanine to L-tyrosine. Zhang et al. (2016) expressed mutant PAH or TPH in *Saccharomyces cerevisiae* to catalyze the production of 5-HTP from L-trp. The activities of PAH and TPH in the hydroxylation of trp with MH_4_ and BH_4_ as cofactors were compared. The results showed that the hydroxylation activity of TPH using BH_4_ as a cofactor was 17 times higher than that of PAH using MH_4_ (Zhang et al., 2016).

With an in-depth understanding of the biosynthetic pathway of BH_4_, Yamamoto et al. (2003) synthesized BH_4_ through the heterologous expression in *E. coli*. The synthetic pathway of BH_4_ is shown in Figure 2. The production capacity of GTP (the precursor of BH_4_) was increased by mutation breeding (Perkins et al., 1999). By optimizing the activity of GCHI (the enzyme that catalyzes the first step of BH_4_ biosynthesis) from different sources, 4 g/L BH_4_ was obtained under fed-batch fermentation (Yamamoto et al., 2003).

Kino et al. (2009) first expressed the mutagenic TPH in *E. coli* and synthesized 0.8 mM of 5-HTP by adding BH_4_ as a substrate. Subsequently, the BH_4_ regeneration pathway and glucose dehydrogenase from *Bacillus subtilis* were introduced to increase the utilization rate of BH_4._ The yield of 5-HTP increased to 2.5 mM under these conditions (Table 1) (Kino et al., 2009; Hara and Kino, 2013). Knight et al. (2013) introduced the mammalian BH_4_ synthesis pathway and regeneration pathway into *E. coli* and co-expressed it with rabbit-derived TPH1. When L-trp was used as the substrate, the yield of 5-HTP reached 198 mg/L (Knight et al., 2013). Although 5-HTP synthesis was achieved by introducing the synthesis and regeneration pathway of BH_4_, the yield of 5-HTP was low and additional L-trp was needed, indicating that it is not a suitable method for mass production.

**Table 1:** Overview of 5-HTP production by microorganism.

**Strains**	**Modulations**	**Titer (g/L)**	**Cultivation**	**References**
*E. coli*	Overexpression of mutant PAH,	0.1762	Shake flask; Supplementation of BH_4_ and 5 mM L-trp	Kino et al., 2009; Hara and Kino, 2013
*E. coli*	Overexpression of mutant PAH; Insertion of BH_4_ regeneration pathway; Insertion of glucose dehydrogenase from *Bacillus subtilis*	0.55	Shake flask; Supplementation of 5 mM L-Trp	Hara and Kino, 2013
*E. coli*	Mutation of PAH from *Xanthomonas campestris*; Co-expression of MH_4_ regeneration pathway and L-trp synthesis pathway	0.1529	Shake flask; Supplementation of glucose	Lin et al., 2014
E. coli	Mutation of the PAH from Xanthomonas campestris; Co-expression of MH_4_ regeneration and the L-trp synthesis pathway; Transformation into an L-trp high-yield strain	0.962	Fed-batch	Mora-Villalobos and Zeng, 2018
*E. coli*	Mutation of phenylalanine-4-hydroxylases (P4Hs); Deletion of the *pheA, tyrA*, and *tnaA* genes	1.1–1.2	Shake flask; Supplementation of 2 g/L L-Trp	Lin et al., 2014
*E. coli*	Mutation of AAAH; Insertion of the human BH_4_ regeneration pathway; Disruption of tryptophanase	0.55	Supplementation of 1 g/L L-Trp	Mora-Villalobos and Zeng, 2017
*E. coli*	Expression of a truncated human TPH2; Reconstitution of the BH_4_ synthesis and regeneration pathway; Modulation of the plasmid copy number and promoter strength; Modulation of different modules' expression levels	1.3	Shake flask; Glycerol as carbon source	Wang et al., 2018
*E. coli*	Same as the previous line	5.1	Fed-batch; Glycerol as carbon source	Wang et al., 2018
*E. coli*	Expression of a truncated human TPH2; Reconstitution of the BH_4_ synthesis and regeneration pathway; Modulation of the plasmid copy number and promoter strength; Modulation of the relative expression levels among different modules; Designing promoter strength to increase tryptophan production	1.61	Shake flask; Glycerol as carbon source	Xu et al., 2020

### Optimization of trp Supply

5-HTP yield can be increased by increasing TPH activity and introducing BH_4_ synthesis and regeneration pathways. Mora-Villalobos and Zeng (2017) mutated the aromatic amino acid hydroxylase of Cu*priavidus taiwanensis* and obtained the trp preference mutant enzyme *C*_*t*_AAAH-W192F. The mutated enzyme was co-expressed with the BH_4_ regeneration pathway in *E. coli*. Additionally, 5 mM trp could be transformed into 2.5 mM of 5-HTP by shaking flask culture for 24 h. After converting this pathway into L-trp producing bacteria, the synthesis of 5-HTP from glucose was realized. The flask yield was 100 mg/L in 60 h, and the batch fermentation yield was 962 mg/L (Mora-Villalobos and Zeng, 2018).

Wang et al. (2018) introduced human TPH into *E. coli* BL21, and co-expressed it with the synthetic and regenerative pathways of human BH_4_. The engineered bacteria hydroxylated 2 g/L trp to produce 1.24 g/L 5-HTP. The authors further introduced the trp synthesis pathway into the recombinant *E. coli* to realize the biosynthesis of 5-HTP. After the optimization of culture conditions, the yield of 5-HTP was further increased by 13 times to 314.8 mg/L. After module optimization, which included: (a)enzymatic modification to improve the hydroxylation activity of TPH, (b)reduction of the copy number of the trp synthesis gene, and (c) regulation of the promoter strength of genes involved in BH_4_ synthesis and regeneration, the yield of 5-HTP in the modified recombinant strain HTPL01-LMT was 1.29 g/L, 3.1-fold increase (Wang et al., 2018). To improve the stability of this system, the authors further integrated the L-trp biosynthesis pathway into *E. coli* genome and designed the promoter strength of the enzyme-coding gene, which catalyzes the first step of L-trp biosynthesis. To regulate the production of 5-HTP, they also regulated the copy number of the L-TPH coding gene plasmid. After these optimization steps, the amount of 5-HTP of shake flask fermentation increased to 1.61 g/ml, which was 24% higher than that of the original strain (Table 1) (Xu et al., 2020).

## Prospects

This review assesses the synthesis and research progress of 5-HTP. Compared with natural product extraction and chemical synthesis, biosynthesis has the advantages of a short cycle, continuous production and mild reaction conditions; thus, it has garnered considerable research attention. In the biosynthesis process, three aspects of optimization have been implemented to increase the output of 5-HTP; (a) improving the hydroxylation activity of the TPH enzyme by directed evolution, (b) introducing BH_4_ synthesis and the regeneration pathway, (c) introducing the trp synthesis pathway. To date, the yield of shake flask culture has reached 1.61 g /L.

Although 5-HTP production has increased 10-fold, remains insufficient for large-scale commercial production. The metabolic network is a complex system, and the efficiency of the target metabolic pathway is often affected by other metabolic pathways. Therefore, strain evolution and breeding for global metabolism may effectively improve the yield of the target product. Besides, high-density cell culture is another strategy for increasing the yield. Combined with the optimization of fermentation conditions and the improvement of cell culture density, the yield of 5-HTP could be further improved.

## Author Contributions

X-XL wrote the manuscript. BZ and L-ZA revised the Metabolic engineering strategy for 5-HTP synthesis section. All the authors contributed to the literature collection and data analysis, and approved it for publication.

## Conflict of Interest

The authors declare that the research was conducted in the absence of any commercial or financial relationships that could be construed as a potential conflict of interest.
